# Epigastric pain associated with herpes esophagitis: case report

**DOI:** 10.1186/s12879-020-05487-5

**Published:** 2020-10-14

**Authors:** Nabil Antaki, Ziad Aljarad, Howayda Dabbas, Walid Haddad, M. Amin Akil, Ahmad Sankari Tarabishi

**Affiliations:** 1 Gastroenterology Department, Aleppo, Syria; 2 Department of Internal Medicine, Faculty of Medicine, Aleppo University Hospital, University of Aleppo, Aleppo, Syria; 3 Pathology Department, Aleppo, Syria; 4 Faculty of Medicine, University of Aleppo, Aleppo, Syria

**Keywords:** Herpes, Esophagitis, Case report

## Abstract

**Background:**

Herpes esophagitis is uncommon disease caused by Herpes simplex virus (HSV). While the disease most often occurs in immunocompromised patients, including post-chemotherapy, immunosuppression with organ transplants, and in AIDS, Herpes esophagitis can also occur in immunocompetent individuals.

**Case presentation:**

We report a case of herpes esophagitis in a 72 year- old woman who was presumed to be immunocompromised following prolonged radiotherapy and chemotherapy for lymphoma. Her main symptom was epigastric pain.

Upper endoscopy showed multiple rounded ulcers in lower esophagus. The diagnosis was confirmed histologically by multiple biopsies. The patient received Valacyclovir for 2 weeks and started to get better within 3 days of treatment.

**Conclusion:**

Although there are few published cases of Herpes esophagitis disease in the medical literature, we recommend that this disease should be considered as one of the differential diagnoses when assessing immuno-compromised patients presenting with non-specific abdominal symptoms.

## Background

Although herpes infection is common even in immunocompetent Individuals, herpes simplex esophagitis (HSE) is uncommon.

Historically, most cases of Herpes esophagitis were diagnosed at postmortem examination of immunocompromised or severely debilitated patients [1–3].

However, with the development of diagnostic procedures, many well-documented cases of herpes esophagitis have also reported in healthy patients at risk [4, 5].

What makes Herpes esophagitis clinically important is that herpes simplex esophagitis can overlap with reflux symptoms, leaving many HSE patients undiagnosed [6].

The symptoms previously published in the literature are acute onset of esophageal complaints such as chest Pain, odynophagia, dysphagia for both solids and liquids, heartburn and/or vomiting [7], whereas no specific symptoms were presented in 26% of patients [8].

We describe here a case of immunocompromised woman due to chemotherapy and radiotherapy for lymphoma, presented with non specific symptoms like epigastric pain, not associated with diarrhea or vomiting and not related to food. Her endoscopic and histological findings were consistent with HSE.

## Case presentation

A 72 year-old women presented to Emergency complaining of epigastric pain that started 3 days ago, the pain was an isolated upper gastrointestinal symptom, not associated with diarrhea or vomiting and not related to food or any other triggers, this episode of pain was not the first one.

The patient suffers from hypertension, ischemic stroke, a treated lymphoma with chemotherapy and radiotherapy 6 years before, and a vertebral compression fracture in T12. There was no history of oral herpes.

Her SaO2 was 99%, Tempreture 37.5 C, Pulse: 100/Rog, Blood pressure: 100/70 mmHg.

On Physical examination, she was conscious,oriented, and her general condition was good, she was not pale, no cyanosis were noticed, her heart sounds and chest were normal, and no edema was noticed.

On abdominal exam, she had epigastric tenderness only and Murphy sing was negative. There were no palpated masses.

Her Lab tests were: (Hemoglobin: 11.3 g/dl) (Hematocrit 38.7) (Red Blood Cells 4.63 cell/ul) (White Blood Cells 14,800 cell/ul) (Platelets 314,000 cell/ul) (C-Reactive Protein 2 mg/l) (Creatinine 1.4 mg/dl) (Urea 67 mg/dL) (Alanine Transaminase (Alt) 12 (U/L) (Alkaline phosphatase (ALP) 71 (U/L) (Amylase 160 (U/L).

On radiology investigations, ultrasonography revealed no abnormal findings, the liver, gallbladder, and spleen all were normal, no masses or cysts were noticed in pancreas.

**Fig. 1:**
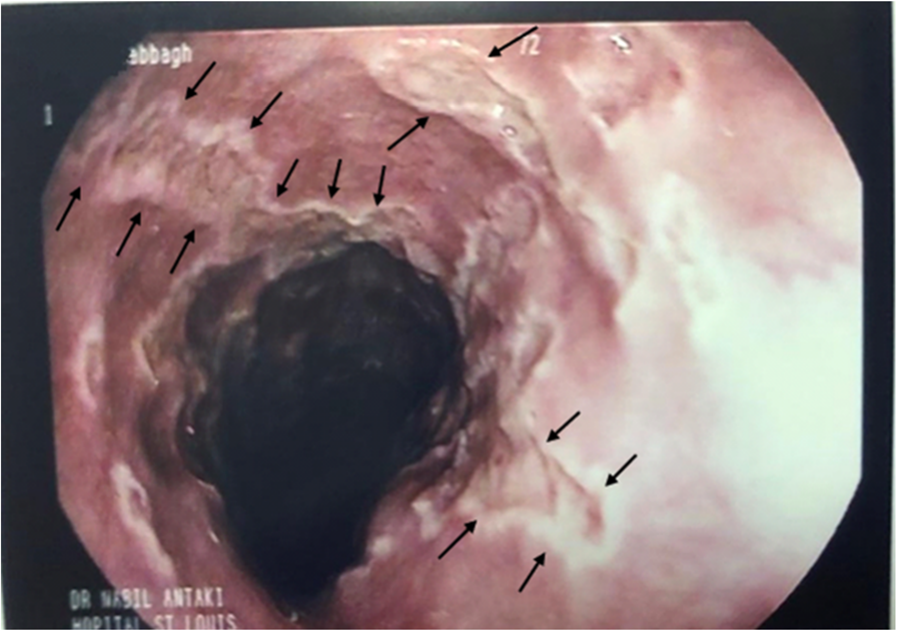
Upper endoscopy and the arrows refer to multiple rounded ulcers in lower esophageos

**Fig. 2:**
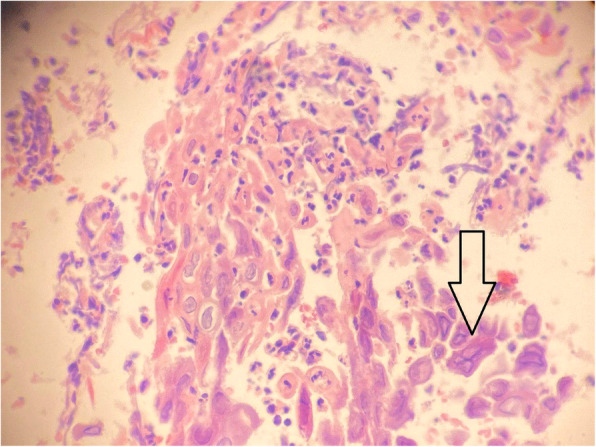
Pathology examination, and the arrow refers to enlarged nuclei and multinucleation

We performed upper endoscopy and found multiple rounded ulcers in the lower esophagus (Fig. 1) and sent a biopsy for pathology examination that showed fragments of squamous mucosa with massive neutrophilic and eosinophilic exocytosis, enlarged nuclei and multinucleation (Fig. 2), there were no herpetic lesions in oral cavity, pharynx, upper and middle esophagus, these findings suggested acute ulcerated esophagitis consistent with herpes simplex viral etiology, no malignancy was in the specimen.

The patient had a symptomatic treatment with intravenous fluids and proton pump inhibitors (PPI), then we applied Valacyclovir 1000 mg twice a day for 2 weeks.

After 3 days of treatment, she started to get better and after 10 days she had no symptoms.

No bacterial or fungal infection was noticed on the herpetic lesions.

## Discussion and conclusion

Herpes esophagitis is usually a self-limited disease in healthy patients or immunocompetent patients, However, it can strongly affect immunologically compromised patients including post-chemotherapy patients [1, 5],organ transplant patients with anti rejection treatment for acute rejection of transplanted organ [6], and HIV patients, it’s a relatively rare clinical condition that affect esophagus which has been reported as the most frequent infection site for HSV-1 and HSV-2.

Herpes esophagitis was first described in 1943, by Pearce and Dagradi, they reported four cases of esophageal ulceration in which intracellular inclusion bodies at autopsy are similar to those seen in viral infections [9].

The reported cases of herpes esophagitis have varied by the method of diagnosis, upper endoscopy “that was first made by Weiden and Schuffler” [10], often shows ulcers throughout esophagus that merge with normal mucosa of esophagus, blood tests for HSV IgM and IgG, PCR, viral tissue culture, but the accurate diagnosis is by obtaining biopsies from esophagus mucosa with microscopic evaluation.

The most frequently clinical symptoms that patients show with herpes esophagitis are dysphagia, odynophagia, upper gastrointestinal bleeding, and hiccups. In this case, epigastric pain was the only feature the patient presented with, and this is not frequently described in the literature.

To diagnose our case, the differential diagnosis of epigastric pain was performed to rule out the common causes of epigastric pain, blood tests and endoscopy had been used for diagnosis, upper endoscopy had shown no signs of PUD [11] in the stomach and duodenum, lab tests of patients serum for pancreatic enzymes was negative which exclude pancreatitis [12], and HIV test was negative which exclude HIV infection as a cause of immunosuppression factor. Upper endoscopy showed multiple rounded ulcers in the lower esophagus, HSV blood tests, PCR, and viral culture were not available, therefore we could not perform them. The definitive diagnosis factor was histologic examination of the esophageal biopsy that revealed fragments of squamous mucosa with massive neutrophilic and eosinophilic exocytosis, the cells showed enlarged nuclei with multi-nucleation. These histological changes are characteristic for viral esophageal infection by human simplex virus.

Our patient was on prolonged steroidal treatment “prednisolone” for recurrent lymphoma,for epigastric pain no per-oral drugs “NPO” was applied, we only applied proton pump “PPI” ,IV fluids, and anti-spasmodic drugs as a first line therapy, after we ruled out the causes of epigastric pain, and histologic examination of the esophageal biopsy confirmed herpes esophagitis, we applied “Valacyclovir1000mg” twice a day for 2 weeks as an antiviral treatment, after 72 h the symptoms of our patient began to disappear gradually.

In our case we found that the patient complained of isolated epigastric pain which it is a new clinical feature for herpes esophagitis, and not discussed in the literature before. Herpes esophagitis maybe caused by prolonged steroidal treatment “prednisolone” for recurrent lymphoma due to its immunosuppressive effect.

## Data Availability

All data generated or analysed during this study are included in this published article .

## Abbreviations

HSV: Herpes simplex virus
PPI: Proton pump inhibitors
PUD: Peptic ulcer disease
NPO: No per oral
PCR: Polymerase chain reaction
Hg: Hemoglobin
CBC: Complete blood count
PLT: Platelets
ALT: Alanine aminotransferase
HSE: Herpes simplex esophagitis
CRP: C-reactive protein
HT: Hematocrit
Alk.ph: Alkaline phosphatase
